# Are we ready for the next pandemic? Lessons learned from healthcare professionals’ perspectives during the COVID-19 pandemic

**DOI:** 10.3389/fpubh.2023.1048283

**Published:** 2023-03-30

**Authors:** Dalal Alsaeed, Abdullah Al-Ozairi, Hussain Alsarraf, Fajer Albarrak, Ebaa Al-Ozairi

**Affiliations:** ^1^Clinical Care Research and Trials Unit, Dasman Diabetes Institute, Kuwait City, Kuwait; ^2^Faculty of Medicine, Kuwait University, Kuwait City, Kuwait; ^3^Ministry of Health, Amiri Hospital, Kuwait City, Kuwait; ^4^Primary Health Care, Ministry of Health, Shamiya Health Center, Kuwait City, Kuwait

**Keywords:** COVID-19, psychological impact, healthcare professionals, resilience, qualitative study

## Abstract

**Background:**

The mental health and wellbeing of people watching the Corona Virus Disease 2019 (COVID-19) pandemic unfold has been discussed widely, with many experiencing feelings of anxiety and depression. The state of mental health of medical staff on the frontlines providing care should be examined; medical staff are overworked to meet the demands of providing care to the rise in cases and deterioration in capacity to meet demands, and this has put them under great psychological pressure. This may lead to an increase in medical errors, affect quality of care, and reduce staff retention rates. Understanding the impact the pandemic has had on healthcare professionals is needed to provide recommendations to prepare for future crises.

**Objectives:**

To be able to meet the needs of the medical workforce on the frontlines and inform psychological support interventions and strategies for future pandemics, we aim to identify and explore the psychological impact of COVID-19 in Kuwait on healthcare professionals in close contact with patients.

**Methods:**

Using semi-structured interviews, we conducted interviews between February and July 2021 with 20 healthcare professionals across Ministry of Health hospitals who were part of COVID teams. Interviews were transcribed verbatim, and analysis was conducted using principles of thematic framework analysis.

**Results:**

Three themes emerged to help prepare future healthcare frontline workers on an individual, organizational, and national level: enhance self-resilience, a better-equipped workforce and healthcare environment, and mitigate stigma and increase public awareness.

**Conclusion:**

The results have assisted in highlighting areas of improvement to support the healthcare workforce in the current environment, as well as better prepare them for future pandemics. The findings have also provided insight to recommend targeted interventions. These should improve the psychological wellbeing and help in supporting healthcare professionals to reduce burnout, continue effective care of patients, and enhance resilience.

## 1. Introduction

Since the end of 2019, the emergence and spread of the SARS-COV-2 virus has reached pandemic proportions. What began as a fairly contained outbreak in the city of Wuhan in China has now been classified as a worldwide pandemic, with trends in cases and deaths increasing alarmingly. The Corona Virus Disease 2019 (COVID-19), caused by the SARS-COV-2 virus, is characterized by severe acute respiratory symptoms. Severe symptoms may lead to death as a result of respiratory failure (1, 2). A variety of public health and social measures have been put in place and regularly updated to limit the spread of this insidious virus, but the uptake of these measures have not been consistent worldwide, with countries adopting various combinations at different times. Curfews, social distancing, relegating work to home, and virtual education are examples of such measures, which have been successful in gradually decreasing the trend of new cases and deaths. According to the World Health Organization (WHO) there have been 755,703,002 confirmed cases and 6,836,825 deaths worldwide attributed to COVID-19, as of 13^th^ February 2023 (3).

The psychological impact of COVID-19 has been significant worldwide. The mental health and wellbeing of people watching this pandemic unfold and those forced to stay at home has been discussed widely, such as experiencing feelings of anxiety and depression (4–7). Pooled data from a review and meta-analysis of 50 studies showed that the highest morbidity was poor sleep quality (40%), followed by stress and psychological distress (34%), anxiety and depression (26%) (8). Pooled prevalence rate of psychological morbidities with respect to impact of event due to COVID-19 pandemic was 44% (95%CI: 42% to 47%). The burden of these psychological morbidities was highest among COVID-19 patients, followed by healthcare workers and the general population. The prevalence of depression is 7-times higher than what was reported in 2017, indicating the huge impact COVID-19 has on people’s mental health (9).

The prevalence of mental health disorders in healthcare professionals (HCPs) has been on the rise, even prior to the COVID-19 pandemic (10). Studies have also shown the dire impact that previous epidemics and pandemics have had on the psychological wellbeing of HCPs, with acute stress disorder, depression, anxiety, burnout, and post-traumatic stress disorder being experienced (11–14). The COVID-19 pandemic has compounded on the already fragile mental health of HCPs, worsening their quality of life and leading to burnout, which has detrimental effects on patient care (15). Professional burnout occurs when staff experience chronic stress in the workplace (16), and this has been heightened further by the COVID-19 pandemic (17). A recent systematic review and meta-analysis looking at the impact of the COVID-19 pandemic on HCPs’ mental health found that 32% suffered from post-traumatic symptoms, 33% from depression, 37% from burnout, 40% from acute stress, 42% from anxiety, and 42% from insomnia (18). A variety of risk factors that can negatively impact HCPs mental health have been identified during the pandemic, such as the lack of personal protective equipment (PPE), concerns about their family’s welfare, and fear of contagion (19).

In Kuwait, earlier cases were associated with imported cases only, but this was soon overtaken by cases of local transmission due to people not following strict preventative measures put in place. To tackle this, some areas have been cordoned off and the government updated measures regularly during the course of the pandemic. As of February 13^th^ 2023, Kuwait has seen a total of 662,858 cases and 2,570 total deaths since the start of the pandemic. At the peak of the pandemic, the Ministry of Health (MoH) called upon HCPs working in the private sector to assist in fighting this disease in order to meet the rising demands. Prior to the pandemic, there were many resignations of HCPs, with many more following at the height of the pandemic due to burnout; this led to shortage of staff and an increase in workload on those still working.

One aspect that requires further attention is the state of mental health of medical staff on the frontlines providing care to those suffering from COVID-19; this needs addressing on multiple levels to mitigate against long-term effects (20, 21). Medical staff are overworked to meet the demands of providing care to the rise in cases, and this has put them under great psychological pressure (22–25) draining them both physically and emotionally. The rates of depression and anxiety in physicians in China and across the world have skyrocketed (23, 26) and being in quarantine is the highest predictor for acute stress disorder (6).

With the upsurge of new cases and the deterioration in capacity to meet these demands in Kuwait during the pandemic, patients and medical staff suffered from psychological pressure. The impact of psychological distress on HCPs tackling this disease may be dire, and may increase medical errors, affect quality of care, and reduce staff retention rates. Burnout in HCPs working in Kuwait has been found to be alarmingly common (27), and it is imperative to identify how this has been compounded due to the pandemic. Burnout has physical and emotional manifestations, such as mental and physical exhaustion, feelings of disillusionment, anger, headaches, and hypertension (28). In addition, HCPs in Kuwait reported high levels of anxiety and depression (29, 30). There are many underlying rationales for the rise of mental health issues; fear and uncertainty leading to irrational behaviors, and peoples’ altered perception of risk due to the delay of detection and the novelty of the virus have contributed to this rise (6). Furthermore, there was a shortage of adequate PPE and of investigations and treatment. The media also played a pivotal role, with important local figures disparaging the efforts of the MoH in Kuwait, and by extension the HCPs involved, for example spreading reports about incidents at different hospitals, while the HCPs were striving to do their best. Fearmongering and misinformation were rapidly spread, and this therefore increased anxiety; examples of this in Kuwait were the spread of messages about various herbal cures, the use of hydroxychloroquine as a treatment, how people were contracting the virus and dying, and the shortage in swabs for testing.

Although there were previous epidemics in neighboring countries, such as the Middle East Respiratory Syndrome (MERS) in Saudi Arabia (31), there was little impact of this in Kuwait. The COVID-19 pandemic would be considered the first major crisis experienced by HCPs in Kuwait. There is a need to fully understand and explore the psychological impact the COVID-19 pandemic had on frontline medical staff; these were unprecedented circumstances where difficult decisions had to be made in an ever-evolving environment and psychological support is essential. By identifying and exploring how COVID-19 impacted HCPs in Kuwait, informed targeted support can be recommended and plans put in place to prepare the healthcare workforce for ongoing demands of working in the healthcare context, and for times of additional demands such as a future pandemic response.

## 2. Methods

Qualitative methods using semi-structured interviews were chosen. Purposive sampling was used to fulfil our objectives; physicians who were directly in contact with patients diagnosed with COVID-19 who were being provided care in MoH hospitals were eligible. Physicians were approached by AO, who is a neuropsychiatrist, and his team of psychiatrists; Kuwaiti and non-Kuwaiti physicians were included to provide a diverse perspective on experiences. Potential participants were provided information about the study by the interviewers; this was then followed by the consent procedure. Recruitment continued until data saturation was reached; iterative analysis ensured this was achieved (32).

The MoH hospitals are public sector hospitals which are nationally funded and provide care to all catchment areas; sampling was conducted from all general hospitals which cover internal medicine and had the most influx of COVID-19 patients. This ensured a representative sample across national hospitals.

Interviews were chosen rather than online questionnaires as they elicit in-depth information from participants. Interviews were either conducted through video-calls *via* the Zoom application to overcome any quarantine regulations, or through audio-recording. Zoom for Healthcare is a cloud-based video-conferencing tool with the ability to securely record and store content as aligned with the Health Insurance Portability and Accountability Act (HIPAA) standards. This tool has been used in other qualitative healthcare research successfully (33). Using video over regular phone calls was chosen as this allows the interviewer (a psychiatrist) to observe their facial expressions to add context, and be able to respond to their concerns accordingly, such as any psychological distress felt during the interview. Limitations of this data collection method may include technological issues and a possible negative impact on establishing rapport (34), although other work comparing it with different methods found it simpler and more convenient (33).

Participants may encounter distress when reliving their experiences during the interview; this was mitigated as the interviewer is a psychiatrist who is able to address any distress accordingly. As the interviewers are psychiatrists who are part of a telepsychiatry service, participants can call at any time to discuss any further issues that may arise later. Participants were reassured that all information is kept anonymous and confidential to protect their identity.

Data protection was guaranteed through various measures. The interview recordings were saved on one account, which is a hospital-provided account under AO’s name; internal access was only provided by him. The video files were recorded on one hospital computer and saved locally on the hard drive secured by the hospital network under all the privacy laws of the MoH. This was shared with the team member undertaking transcribing, who has undergone Good Clinical Practice training and understands the importance of patient confidentiality and data protection. Ethical approval was sought from the MoH, as participants are based in MoH hospitals (approval number 1534/2020).

A semi-structured topic guide was developed in both Arabic and English. This has been informed by previous research (7, 22, 23, 35) and discussions with HCPs. Interviews took place between February and July 2021. The interviews were conducted in either Arabic or English, depending on the participants’ preference. Interviews were transcribed verbatim by a member of the team. The data was managed using the qualitative data management software MAXQDA 18 (36). Initial analysis was performed by DA, an experienced qualitative researcher, and discussed with the team. Interviews were transcribed in the source language to reduce the risk of translation incongruities that may impact data analysis (37).

There are diverse approaches to qualitative analysis, with thematic analysis underpinning them (38). Compared with other qualitative analysis methods, thematic analysis is seen as a flexible technique that can ‘provide a rich and detailed, yet complex, account of data’ (38). Analysis was conducted using principles of thematic framework analysis, and it was an iterative process. When using thematic framework analysis, it does not necessarily mean relying on the deductive approach, as there is flexibility to this analysis method, and both deductive and inductive approaches can be used to answer the research questions (39, 40). The framework approach consists of 3 stages; data management, descriptive accounts, and explanatory accounts, all in a continuous process (41).

Both inductive and deductive approaches were utilized, as well as constant comparison across transcripts; this was to ensure that theories are not limited to what is already known, and that analysis is not rigid (42). Constant comparison enabled an iterative approach whereby themes were searched and compared across participant data sets (43, 44).

Reflexivity in qualitative research is integral towards establishing rigor (45). The first author, AO, is a neuropsychiatrist who led the interviews with his team of psychiatrists; two interviewers are Kuwaiti and one is Egyptian; the Egyptian interviewer led the interviews with the non-Kuwaiti participants. As the healthcare profession is a tight-knit community, some of the participants were known to the interviewers; although this may pose some bias, it provided a conducive environment for the interview as participants felt at ease and discussed personal topics.

## 3. Results

### 3.1. Characteristics of participants

Twenty physicians were interviewed; characteristics of the sample are reported in Table 1. All work at large public hospitals, six in total. Quotes are followed by a code assigned to each participant, and each hospital is denoted with a code to safeguard anonymity of participants.

**Table 1:** Characteristics of participants.

	Number of participants
**Gender**	
Male	13
Female	7
**Job title**	
Medical Trainee	1
Medical Assistant	2
Assistant Registrar	3
Registrar	7
Senior Registrar	3
Consultant	3
Specialist	1
**Age (years)**	
25–30	6
31–35	4
36–41	10
**Nationality**	
Kuwaiti	10
Non-Kuwaiti	10
**Contracted COVID-19 virus before interview**	
Yes	8
No	12
Total number of participants	20

The themes that emerged from analysis of the data include: (1) enhance self-resilience, (2) a better-equipped workforce and healthcare environment, (3) and mitigate stigma and increase public awareness.

### 3.2. Enhance self-resilience

Participants discussed the trajectory of feelings, emotions, and experiences they had as the pandemic unfolded. A variety of psychosocial issues were experienced; depression and anxiety, trauma, stigma at work, isolation, burnout, feelings of guilt, and dealing with grief were all felt by the participants. Those with families had fears of contagion and spreading the virus to their loved ones; this led to many isolating themselves from their families, which had a toll on their mental health.

“Then I felt soo horrible, I mean I’m confined in a room, I have a bathroom, I have everything I want, my food comes to my doorstep but I can’t see my daughter! I can’t hug her! And I cannot see my family! I hear her coming across the corridor to go to our bedroom and sleep, and she asked ‘where’s daddy where’s daddy?’ ‘And daddy’s not there!’ That period was reaaally really difficult emotionally to cope with that isolation.” COV04, BC

Everyone suffered from some form of isolation, whether it was at work and being shunned by other colleagues not part of the COVID teams, or not being able to return home to be with their families as they feared for their safety. Some felt this isolation acutely as their families were back in Egypt. This negatively impacted the participants’ mental health, with some experiencing depression and anxiety as a result, and one participant attributing to it having a wider impact on his current behavior.

“Of course, thank God everything is different now…but I’m still affected…I mean I feel that in the last few months, something has changed in me which isn’t good…I’ve become prone to isolate myself from others…” COV20, B

There was also social stigma with participants reporting friends refusing to meet them due to their work with COVID and fearing getting infected; participants felt upset and this also led to isolation in some cases.

“At the beginning, the COVID team were like exiles, you know? We were isolated at work, almost no one talked to us or said hi, and if anyone saw us walking nearby, they would rush the other way, and this was whether we were wearing hazmat suits or not” COV09,B

Many of the participants expressed how they underwent an internal struggle with a dichotomy of emotions; they felt it was their responsibility to care for patients but on the other hand felt guilty of risking getting infected and passing it on to their loved ones, and some felt guilty for putting themselves before their patients when having to don PPE before helping them.

“it’s hard because you have a responsibility, you’re seeing patients and sacrificing for them but you have to think about your family at home and you might infect them or worse lose a loved a one!! You have to think this is not for me, but for my family…and you’re in this internal struggle with yourself…between your duty and your fear for your family and children and parents…it’s really hard! It’s a huge responsibility!” COV06, E

They also experienced low self-esteem and feelings of helplessness and guilt as no matter what they did, their patients deteriorated or died.

“Yeah! I definitely felt like an accomplice, like I was associated with that, like I was a part of that. That’s why one day I said I can’t be here anymore, I didn’t want to be a part of this, I feel like it’s affecting myself, and I felt more injured, every day I wake up and go “How can I call myself a doctor!?” you would say but you are saving lives! I’m not saving a single life! Every day where I sit, I come to work, I don’t see the patient get better because of me, they get better despite of me! That’s how I felt sometimes.” COV07, BC

Some participants had a family member pass away from COVID with them not being able to see them due to their working conditions; this played a role in their guilt. All experienced a sense of duty and ethical obligation to be part of the COVID team, warring with feelings of despair and fear. Some attributed this internal struggle to feeling guilty if they did not help lessen the burden on their colleagues. All these psychological issues were reflected in the participants’ personal lives and their families.

“But…it affects me when I hear that one of my colleagues passed away from COVID…this is when I realize how scary the situation is and it hits home…and from time to time I get black thoughts…what if something happened to me and my kids are so young…but then I quickly stop my train of thoughts and tell myself this is my job, yes this is my job…and we swore an oath! We swore an oath and we must respect that!” COV06, E

It was evident from the discussions that participants sometimes found it difficult to see that the daily psychological pressure was leading to a gradual deterioration in their mental health, with some experiencing depression and burnout without realizing. HCPs lack the skills and knowledge needed to identify what they are experiencing; by providing *psychological training* to accomplish this, HCPs can seek psychological help in a timely manner.

“It requires skills to deal with burnout, and at the same time the physician should be able to identify that he’s experiencing burnout, and that it’s normal, and that the problem is not with you, the problem is how you’re managing the situation. This is really important!” COV03, A

Participants discussed how they lacked the *psychological support to deal with psychosocial issues.* Participants supported the use of psychological interventions, which were currently lacking. One-on-one and group therapy was suggested, as well as regular screening for depression and anxiety. Even the chance to talk about their experiences, such as the interviews conducted, was deemed beneficial.

“Yes, I think they would have benefited from psychiatric or psychological help. Because really everyone was stressed and everyone had fears and were upset because of all the changes that were happening, I mean other than the pressure at work, we all had our lives upended you know? I feel like every time I spoke with a colleague, or actually every time a colleague spoke with me I began to realize that we were all upset…everyone is going through negative emotions and I think it would have been a good idea if some help from the mental health team would have been provided.” COV05, D

To cope with the overwhelming stress of working in COVID teams, participants described some of the adaptive *coping mechanisms* they used. Some participants self-reflected and meditated, while others relied on praying and resorted to religious coping to provide peace of mind and to feel “human.”

“We tried as much as we can to focus on spirituality…I mean I used to listen to some religious lectures, and these helped me…you feel like a deity and you start losing your humanity…increase oxygen, reduce oxygen…” COV19, B

Others described the activities they did, such as playing video games or binge-watching TV series, as things they used to do in their childhood that used to bring them joy.

“I began distracting myself with other things, things I stopped doing for a long time…like for example I started watching…before you’d have hobbies or things you liked doing, like listening to certain songs, music, watching series…as we got older, we forgot these interests and got caught up in work and had bigger responsibilities. Honestly, I started living my teenage years again!” COV01, A

Focusing on “human” tasks at home, such as laundry and cleaning, helped one participant as he felt he could clean his mess and control the outcomes; this reflects on how chaotic the experience with COVID was where HCPs felt powerless. Many avoided the media and tried to separate from their work life when they got home by “switching off” and sleeping. Some used the opportunity to focus on continuing their education as a distraction. When HCPs tested positive and had to self-isolate, many found it difficult to cope and lacked the strategies to detach from their current experience. Others found self-isolation as an opportunity to discover past interests that helped them cope.

“Isolation had a positive effect on me in one respect, in that some interests that I gave up a long time ago because of family responsibilities and other things, such as reading literature far from the medical field, writing, things like this…I spend 3-4 hours and go back to reality, and it’s like a reset for the mind, takes you away for the medical field for a bit and brings you back.” COV10, B

Coping strategies adopted by the participants were all forms of individualized self-care, but they all achieved the same goal of supporting participants in adjusting to this new and ever-changing environment and to reduce the impact this had on their mental health and enhance their resilience.

*Social support*, whether it was from the participants’ families or the community, was not always available, and this further exacerbated their psychological wellbeing. Not all had supportive families, with some putting pressure on them to leave the COVID team. Others reflected on the lack of support from the public and their social circles. Some stated that even within their immediate environment and team, they would receive negative feedback and energy which was detrimental to their coping in the long run. Some participants had a support system in place, such as friends and colleagues that they could talk to or had access to a psychiatrist.

The healthcare force is made up of expatriates, the majority of whom left their families back home. Although some were grateful their families were not with them as they feared for their safety if they brought the infection home, being alone during this stressful time compounded their isolation and it was damaging their mental health, especially as they were unable to travel back home.

“Before COVID, I would go back home every 6 months or my family would come over…but the feeling that you were trapped and you’re helpless if anything happened to your family back home…and I’ve seen this happen with my colleagues, and that’s a horrific feeling, it’s indescribable!” COV02, A

One of the participants was pregnant during the first wave and many people criticized her. This intensified her guilt of putting herself and her unborn baby in danger, adding to her already growing stress and anxiety.

While the majority of people were working from home during the pandemic, HCPs were continuously working to keep everyone safe throughout the pandemic. One area that was lacking support was schooling; female participants with young children were working long shifts and having to manage online schooling, a completely new experience, alongside all the issues faced with the pandemic.

“We work till 1, and this is without our on-call and our normal work, and the online [schooling] is during our working hours, even when I requested afternoon schooling, they just started 11:30/12, so how can I make it back home?? I know I’m a doctor but I’m also a mother…and I would have to sacrifice something…it was hard…this is my children’s future, my son is in his foundation years in first grade…this made the situation so much more difficult.” COV06, E

Appreciation and support for HCP efforts, in the form of thank you tokens from patients and food donated by local businesses had a big impact on bolstering HCPs’ wellbeing.

“There was a restaurant which made a pledge ‘these people [HCPs] are working hard and need to eat well’, and that felt amazing, it was a great gesture…that people would offer social support for the medical teams.” COV12, C

The participants all described the psychological impact the COVID-19 pandemic had on their mental and physical wellbeing; enhancing HCPs’ self-resilience to better manage the psychosocial factors should be deemed a priority to prepare them for crisis-management on an individual level and ultimately prevent burnout. This can be achieved through psychological training, readily available and accessible psychological support, targeted interventions to support coping, and social and family support.

### 3.3. A better equipped workforce and healthcare environment

On an organizational level, in terms of *working conditions, environment, and healthcare management*, many aspects came to light during the discussions that had an adverse impact and required improvement. With the pandemic, the working conditions within hospitals and the dynamics changed dramatically. HCPs were forced to work in a completely new context, outside of their specialty, and with a new team. Alongside the pressure of treating patients, they had to maneuver and adapt to this new setting and team dynamics, with many struggling to achieve team cohesion. This sometimes led to suboptimal patient care.

“It was a bit of a learning curve, because it was a new hospital, I didn’t know anyone there, completely new team. The nurses; I didn’t know nurses and it took me a while to figure out what nurses knew and what they were capable of…That was a problem. Because it came very clear that they were not the standards that I used to work in [previous hospital]. Here I expect things are done in a certain way and almost becomes automatic there and became very quickly, NO! I would have to do a lot of micromanaging which I don’t like doing anyway! I have to audit everything that has been done and audit every order to make sure it’s been carried true.” COV07, BC

In addition, the long working hours and shortage of breaks and inability to take time off also impacted HCPs physically and mentally. Many reported a shortage of PPE at certain times during the pandemic, which meant they would sometimes sacrifice their own safety to treat patients. The decision-making process regarding treatment was affected greatly as there was a lack of standardized and unified treatment protocols, which also sometimes put HCPs in ethical dilemmas. Participants reported instability in daily policies and procedures regarding admission and discharge of patients, which added to the ever-growing confusion.

“Our problem was with the protocols and management, the management never gave us a chance to develop protocols, and every time we agreed on something, our boss which change everything…and this wasn’t only in Kuwait, but worldwide, there was no consensus on disease management…there’s no set protocol, every day there’s a new recommendation, a new management strategy…this was there problem.” COV02, A

Some hospitals implemented operation protocols that supported healthcare staff, which in turn reduced their risk of burnout and maintained appropriate staffing numbers.

“I didn’t see people falling apart! That’s the other fortunate thing! I haven’t seen people that got so emotionally affected by what’s going on! And I feel part of it, that we were fortunate that we had a controlled admission in general. Part of it, we limited the number of patients we have per physician. When you don’t have to look at a unit of 30 patients, you are the only one who’s rounding on them, it’s different when you are rounding on 15 or maximum 20! And this is well-known even prior to COVID, that certain staffing number is what’s acceptable and what works well! And I felt by maintaining this by us maintaining that, we assured that we won’t have people burning out and falling apart.” COV04, BC

Trauma was also perceived from the setting itself; HCPs had no area to rest and recharge, and in some cases the space provided was not conducive. Some participants suggested that hospitals allocate an area for exercising, for example, to unwind during work.

“There was this horrible and scary office, with dimmed lighting, really dim lighting in a room without any comfortable chairs, with no avenues to have fun at all, just sitting around waiting, just waiting…this greatly contributed to our depression…firstly the situation was difficult, the atmosphere was tense, there was no compensation and no relieving factors…” COV09, B

Some hospitals provided isolation rooms for staff that required them, such as those who did not want to risk infecting their families, and this was an excellent effort to ameliorate HCPs’ stress.

Not all reported support from their management, which played a vital role in their daily work and worsened their stress. Some described how their hospital management encouraged autonomy and provided decision-making support, which alleviated the pressure on them.

“Luckily, things were, the group who gelled very well, stayed together in the hospital, the head of department didn’t want to interfere in the daily work but was supportive whenever we needed things to be done, wasn’t dictating how we managed the patient, he left us complete autonomy on how we managed the patients, we were able to increase the number of units over there, things just fell in their place appropriately.” COV04, BC

Appreciation and understanding from hospital management had a profound effect on participants, which saw the impact ripple across staff.

“Honestly, Dr. S and the management group as a whole emotionally supported us, in a big way, ‘we know you’re tired, we know there are problems but please bear with us’, these words made a huge difference to us, 180-degree change from being negative to positive, someone who actually understands you! I began trying to be like them…I mean trying to support each other… ‘guys we’re doing good, we’re making a difference, we have to continue fighting, we are making sure the infection doesn’t spread to ourselves and our homes…we are doing something that others may not see!’” COV13, B

Not being able to take time off work, even for a few days, intensified HCPs’ burnout; better work pattern management and rotating staff may have helped in providing some relief.

*Regular and intensive training for healthcare teams to deal with crises* is needed. Through the discussions with participants, it became apparent that not all teams were at the same standard, and those with experience had to manage and oversee the care.

“So, the first patient was the first one who was admitted to the ICU [intensive care unit] anywhere in Kuwait, got intubated, was difficult to ventilate and oxygenate and received ECMO [extracorporeal membrane oxygenation] within 12 hours of admission to ICU. So, to provide such advanced therapy in a unit that neither physicians nor nurses are familiar with a technology or with the complications if something goes wrong, that was a huuuge huge struggle, and that was something that irked me so much.” COV04, BC

Participants also described the process of informing the patients’ family of their death which was very difficult and was met by backlash from the family; this increased HCPs’ stress and they felt inadequate in dealing with it. Grief and communication counseling training is required to better equip HCPs.

“I’m a surgeon by trait, so I don’t really have a good socio-cultural background! I just don’t! We were trying to do certain things like the regular breaking bad news stuff, sort of scale it up a little bit in trauma and that we spend enough time learning more about mental health for us and for the patients. But in general, nothing compared me for dealing with families here….” COV14, C

It was felt by participants that there was a *lack of psychological support for patients*. Participants stated they were mentally and physically exhausted. Not only did they have to deal with their own mental wellbeing, but they also had to offer psychological support to their patients to compensate for the lack in mental healthcare. This took its toll on HCPs who had to spend time and energy to make their patients feel safe and reduce their fears. One participant experienced this when he had COVID:

“Every few minutes I would measure my heart rate, check my saturation. How would you think a patient with no medical background would feel when they constantly hear people are dying? So all of them, well not all of them but maybe 95% would get depressed, have extreme fear, phobia, I think a lot of people…even God rest him in peace Dr. A, they say one of the things that made his health deteriorate was his fear of death, psychologically he was really affected even though he wasn’t displaying severe COVID symptoms…” COV11, B

On an organizational level, supportive and understanding management, resource availability, standardized working conditions and staff rotation were advocated. Regular and intensive training taking into account the experiences of the COVID teams was also deemed imperative for a better equipped healthcare force. There also needs to be psychological support for patients to mitigate the burden on HCPs on the frontline, as well as grief counselors for families if needed.

### 3.4. Mitigate stigma and increase public awareness

On a national level, public awareness of the pandemic and HCP efforts was not at the forefront. The media played a major part in this. The media was also aggravating the situation, with tensions rising and animosity from the public towards the MoH and the HCPs by association.

“Stigma, mmmmm… Nothing direct, but as in general there’s… I don’t know from what I read from social media and stuff, I feel there’s a lot of hate towards doctors lately…” COV16, F

Videos and messages circulated on social media had a direct impact on patient care and affected HCPs’ efforts.

“I was feeling angry during that period, as it was the same time that a video circulated of an actor dying, and people kept saying it was because of corticosteroids…so we had so many patients, extremely sick with bad saturation levels and they would refuse steroids, refuse to be admitted, refuse to go to the ICU, even though they need it…so it was really frustrating!” COV15, F

Participants discussed their frustration when encountering family members or patients that do not believe in the virus nor vaccination.

“I felt that many people did not understand the situation that we were in…I mean I know…thankfully the majority of Kuwaiti society are educated…it’s very rare in this day and age that you find someone, whether Kuwaiti or non-Kuwaiti who is not educated. Everyone reads…everyone has a smart phone and have easy access for any information, you know? But I started getting upset…with time…I mean especially with the curfews, I felt people started blaming HCPs…it’s your fault…vaccination problems, people are refusing vaccination…people were resisting and saying ‘there was no need for curfews! Corona virus is a lie! The vaccine is a conspiracy!’” COV20,B

A more positive approach should be taken to better disseminate information to enhance public awareness. In order to achieve this and gain patients’ and the public’s trust, efforts should be taken to attain their perspectives. The media should also be involved to tackle this issue from all sides across all platforms to mitigate stigma towards HCPs.

## 4. Discussion

It is evident from the results that COVID-19 has had a significant psychological impact on COVID teams, and it is imperative that we address this. HCPs were working in a new and stressful environment, and this added to their experienced trauma. The overwhelming pressure to keep up with the high rise in cases in sometimes inadequate conditions added to their stress. Many reported internal struggles; responsibility and ethical duty against guilt and fear for safety. HCPs are vital resources for every country. Their health and safety are crucial not only for continuous and safe patient care, but also to mitigate the effects of any outbreak. The findings shed light on the factors contributing to the psychological effect of the COVID-19 pandemic on an individual, organizational, and national level and provided suggestions to support medical staff to deal with current ongoing demands as well as prepare them for future crises. This is adapted from the social-ecological framework (46) to help examine the impact of COVID-19 across different levels, from individuals to systems.

Qualitative studies conducted around the world support the current findings, with psychosocial issues such as isolation and social stigma, depression, stress and burnout at the forefront (35, 47–50). The new and changing working environment and its impact on HCPs during this pandemic was also reported. Communication challenges within teams and across healthcare and policy makers was deemed as a stressor and better support structures should be put in place (51). The stigma perceived from family and the public also posed negative implications (52), which was seen in Kuwait as well. Many described their experiences as learning curves, whether dealing with the unknown manifestation of COVID or navigating the dynamic working conditions and teams. This was echoed by HCPs in Oman who described their learning as a continuous process (53).

Based on our interactions with the participants, HCPs felt like they were going to war and there was a war-like mentality, and as soldiers they felt they were not properly trained or built to deal with this pandemic, and thus their coping strategies were not always adequate. Our current healthcare systems focus on chronic diseases and their treatment rather than on infectious diseases at this mass level. It was evident from the findings that although some coping strategies were similar, they were all based on each individual’s background and stage of deterioration of their mental health, as individuals respond to stress differently (54). Coping strategies have protective effects against the burden of the pandemic, and these are recommended to be emotion-based, such as religious coping, with an active approach, such as seeking social support (55). Interventions and suggestions for coping strategies should be tailored to take this into account to increase their uptake and be successful (51, 56). When it comes to support needs, it was apparent that better psychosocial support was necessary. Dissemination of standardized information and protocols was also lacking, which contributed to the issues encountered by the participants. This was also reported in other countries and highlights the change of pace and possible lack of transparency (52, 57, 58).

Resilience is an important concept in this context as it refines the relationship between perceived risk and potential mental health issues (59). Enhancing health organizations’ efforts, such as through better communication, mitigating HCPs’ stress, and focusing on improving work patterns and conditions all assist in building resilience (57, 58).

HCPs showed a tremendous sense of responsibility and concerted efforts in alleviating patients’ suffering, including working in a totally new context, physical exhaustion due to heavy workloads and PPE, the fear of becoming infected and infecting others, and feeling powerless to handle patients’ conditions. To cope with stressful situations, they identified many sources of social support, defined here as support from family members and friends, and used self-management strategies. They also described how they were able to transcend the difficulties inherent in their unique experience.

By understanding the impact of COVID-19 on the mental health of those taking care of patients with COVID-19, this would assist in developing targeted interventions that improve their psychological wellbeing. This would help in supporting HCPs to reduce burnout, continue effective care of patients, and enhance resilience. The landscape following COVID-19 is ever-changing and there is a need to build resilience and implement supportive interventions to help healthcare systems manage the next pandemic.

### 4.1. Recommendations

Some HCPs may not prefer professional help and would rather talk with a colleague informally, whereas others would place more emphasis on PPE availability to ease their fears and anxiety, and thus HCP input is imperative when developing recommendations (60). Some recommendations that have emerged from the findings focus on three areas to better support medical staff with the ongoing demands of working in the current environment, as well as prepare healthcare in Kuwait for future crises.

On the individual level, there is a wide array of mental health support avenues available for implementation. Ensuring HCPs are well supported and alleviating their psychological distress can in turn positively impact patient safety as well as improve staff retention rates (61), and thus efforts should be made to offer targeted support. Based on the findings and participant experiences, the availability of support groups, having a resident psychiatrist for one-to-one sessions, and mindfulness sessions can all meet their needs. An online mindfulness intervention used by HCPs in Kuwait during the pandemic demonstrated improved mental health outcomes (30). Peer support and mindfulness interventions were also suggested by HCPs in Spain during the pandemic (50). Cognitive-based therapy, whether as individuals or groups, has also been shown to reduce symptoms of anxiety and depression in HCPs who have faced crises (62). Part of the psychological support should also include education and training on recognizing symptoms of depression, anxiety, and burnout, as it was evident HCPs had difficulty identifying this. There should also be an outlet for HCPs to voice their needs and be provided support and time off. Managers can enhance resilience by providing tailored coping approaches for their respective team members and fostering trust and communication. In addition, HCPs on the frontlines are the most important stakeholders as they are seeing the shortfalls of the system and can provide significant insight and help from within; as such, healthcare systems should seek HCPs’ input through the provision of a channel for suggestions. Social support should also be taken into consideration to guarantee all aspects are met, as resilience and social support are essential protective factors against burnout (17).

Looking at the organizational level, there needs to be better resource availability in terms of PPE to alleviate HCPs fears and ensure they feel that the healthcare system cares about their safety. It was evident that staff required regular and intensive training to be able to work in any situation and environment, especially in a crisis, and that this training needs to be standardized so no time or efforts are wasted. This pandemic should be a learning experience where better protocols and triage procedures are developed to deal with any similar outbreaks. Future training development should involve COVID teams whose comprehensive experience can ensure all relevant points are included and to better prepare future HCPs. Hospitals should also endeavor to develop and implement mentorship programs to promote workplace support and solidarity. To make sure HCPs can take time off and working patterns are improved, staff allocation should be optimized, with more staff placed in intensive care units. Better management of overworked staff and implementing sufficient breaks were also advocated in previous work (63). Acknowledgement of COVID teams and their efforts by their management was also deemed important and had a positive impact on them, as seen in China as well (64). There should also be a grief counselor for patients and their families to alleviate the pressure on HCPs. HCPs also play a role in reducing psychological stress in patients and their role needs to be brought to the forefront (65). In addition, offering psychological support for patients reduces the pressure on frontline HCPs (66). Training on communicating news of death to families from diverse cultures should also be provided as HCPs come from different backgrounds and may have difficulties in relaying this information in the appropriate way.

On a national level, governments should strive towards better partnerships with the local media outlets. Governments and health authorities can mitigate stigma and assure that correct and evidence-based health messages are shared in a timely manner (66). Patient and public engagement is also imperative to reduce stigma and ensure public awareness messages are disseminated in the best approach.

### 4.2. Strengths and limitations

To our knowledge, this is the first qualitative study undertaken in Kuwait studying the psychological impact of COVID-19 on healthcare professionals. The sample size may be considered small, but recruitment continued until data saturation was reached (32). Although some of the participants may have been known to the interviewers superficially, this assisted in creating a safe environment where thoughts and feelings were shared due to trust and rapport. Furthermore, the interviewers assured the participants of anonymity and confidentiality. We endeavored to include a representative sample of the frontline workforce in Kuwait by including Kuwaiti and non-Kuwaiti doctors; it would be beneficial to include other HCPs in future research. To ensure participants’ privacy and confidentiality during this sensitive and stressful time, we decided not to utilize video recordings for facial expression analysis.

## 5. Conclusion

The COVID-19 pandemic has put the healthcare force on the frontlines under psychological pressure and findings from the current study have identified these stressors and how to target them on an individual, organizational, and national level. The results have assisted in highlighting areas of improvement to better prepare the healthcare workforce for future pandemics and provided insight to recommend targeted interventions that will improve the psychological wellbeing and help in supporting HCPs to reduce burnout, continue effective care of patients, and enhance resilience. The implications of the findings are wide-ranging on practice and policy, and future work should focus on developing and testing the effectiveness of interventions and support mechanisms. This will assist in mitigating the ongoing psychological impact of the pandemic, as well as prepare them for future crises.

## Data availability statement

The original datasets presented in this article are not readily available because they contain potentially identifying or sensitive personal information. Requests to access the de-identified datasets can be sent to the corresponding author at dalal.alsaeed@dasmaninstitute.org.

## Ethics statement

The studies involving human participants were reviewed and approved by the Kuwait Ministry of Health Ethics Review Board (approval number 1534/2020). The patients/participants provided their written informed consent to participate in this study.

## Author contributions

EO led the study conceptualization. DA led the study design, methodology, funding acquisition, and drafted the manuscript. AA-O led the data collection. All authors contributed to revising the manuscript, approved the submitted version, and involved in data analysis.

## Funding

This study was funded by the Kuwait Foundation for the Advancement of Science (Grant number CORONA PROP 88—PN20-13NO-01).

## Conflict of interest

The authors declare that the research was conducted in the absence of any commercial or financial relationships that could be construed as a potential conflict of interest.

## Publisher’s note

All claims expressed in this article are solely those of the authors and do not necessarily represent those of their affiliated organizations, or those of the publisher, the editors and the reviewers. Any product that may be evaluated in this article, or claim that may be made by its manufacturer, is not guaranteed or endorsed by the publisher.
